# The Mediating Impact of IT Capabilities on the Association between Dynamic Capabilities and Organizational Agility: The Case of the Jordanian IT Sector

**DOI:** 10.1007/s40171-022-00303-2

**Published:** 2022-04-15

**Authors:** Abdulkareem Salameh Awwad, Omar Mohammed Ali Ababneh, Mahmoud Karasneh

**Affiliations:** Department of Management and Marketing, College of Business and Economics, Qatar University, P.O. Box 2713, Doha, Qatar

**Keywords:** IT capabilities, IT infrastructure, Operational coordination, Organizational agility, Technical resources

## Abstract

This study suggests a novel progression to the current research endeavor by investigating the influence of information technology capabilities on organizational agility. More specifically, this study aims to fill the gaps found in previous studies and contribute to the current state of knowledge of this domain by focusing on the mediating role that IT capabilities play between dynamic capabilities and organizational agility. Toward that end, 270 Jordanian professionals working in supply chain management and operational departments were approached. Data were collected via distrusting a structured questionnaire that includes items assessing dynamic capabilities, IT capabilities, and organizational agility. The results demonstrated that IT capabilities significantly and positively mediated the relationship between resource-based dynamic capability and organizational agility. The study has also discussed several theoretical along with managerial implications of the research.

## Introduction

In recent 20 years, modern business organizations are experiencing a highly turbulent environment, where their operations and performance are strongly shaped by a wide range of factors, including technological advancements, complementary obligations, time-to-market pressures, and intense competition (Bondzi–Simpson and Agomor, 2021; Bruque-Camara et al., 2016; Harsch & Festing, 2020). This aggravating environment has elevated pressure on business organizations to continuously adjust and predestine their strategies and operational systems in ways that promote effectiveness and efficiency (Calleja et al., 2018; Chikhale & Mansouri, 2015). Under such circumstances, the most crucial and essential strategy is that organizations should develop their capabilities to willingly sense and respond to external modifications and be agile (Kushwaha et al., 2021; Vaishnavi & Suresh, 2020). Organizational agility is considered as the ambidextrous strategy of a company to react to environmental modifications, while enhancing firms’ capabilities to avail dynamic opportunities and discover new avenues (DeGroote & Marx, 2013; Harsch & Festing, 2020). Here, it is worth stating that viewing organizational strategy through the lens of the dynamic capability theory is a captivating rationale, as agility is described as a strategy essential to maintain firms’ investments that help sustain in the unpredictable and turbulent environment (Bondzi–Simpson and Agomor, 2021; Green et al., 2019; Vaishnavi & Suresh, 2020).

Consequently, the hasty pace of intense universal competition, technological development, and cursory modifications and changes in customer expectations and preferences illustrate that the firm’s dynamic capabilities should have the capacity to detect, implement, and react to the adjustments and modifications within such business environments (Harsch & Festing, 2020; Kim et al., 2011; Kushwaha & Kar, 2020a). Among those confounding factors, information technology (IT) is becoming progressively crucial (Gao et al., 2020; Kohli & Grover, 2008; Kushwaha et al., 2021). Accordingly, IT pioneers have jumped to this change bandwagon to establish immediate and comprehensive collaborative settings, consolidating the collective knowledge and resources of all partner firms in order to constitute a greater IT network resources and capabilities that generate effective response to environmental modifications (Kushwaha et al., 2021; Lowry & Wilson, 2016). This intervention and wider applicability of IT can be analyzed from the infrastructural and value-driven aspects (Han et al., 2017).

More specifically, the issues of infrastructure are typically witnessed in connectivity, compatibility, and hardware (Zhang et al., 2009), while value creation includes the issues in workflow and procedures (Athey & Schmutzler, 1995). Amid the COVID-19-induced restricted workplaces, IT interventions provide infrastructural and value creation services that can be accessed from the remote areas. The eruption of pandemic has intensified the need for organizational agility. Therefore, it has become necessary to devise framework and information systems that foster development agility for the organizations. The demand for products that can be updated by the firmware has been increased, and this has led to the rise in demand of software that can be upgraded according to the customers’ needs (Kushwaha & Kar, 2020a). Therefore, the likelihood of the outbreak of another pandemic has urged firms, governments, and populace to devote further attention to agility and resilience (Batra, 2020). Similarly, e-business proactiveness and collaborative knowledge have a greater positive role in magnifying the effectiveness of organizational agility to response to the COVID-19-led crisis (Al-Omoush et al., 2020).

Nevertheless, since the last decade, scholars have been extremely attracted toward adopting the dynamic capabilities view (DCV) in there conceptual models due to the alteration in the market needs and necessities, which is considered as an extension of resource-based view (RBV). Firms need to develop dynamic capabilities for creating, extending, and modifying the ways to enhance their opportunities to survive and prosper under challenging environments (Harsch & Festing, 2020; Helfat et al., 2007). The advancement of theoretical arguments has decomposed the dynamic capabilities of IT sector into a series of identifiable and specific routines, rather than making it an elusive concept (Zhan et al., 2018).

One crucial aspect contributing to the effectiveness of managerial interventions in turbulent environments and managers’ competence in directing a preferred agility/efficiency nexus is a better understanding of how to distinguish between uncertainty and risk (Bondzi–Simpson and Agomor, 2021; Candace et al., 2011). Organizational agility cannot be evaluated independently from a consideration of uncertainty, budgets, costs, risk, strategy, and commitment (Kozioł-Nadolna, 2020). A general framework becomes a prerequisite for managers in grappling complex and interdependent issues effectively. In the IT industry, dynamic capabilities can be separated analytically from the strategic formulation, but must be linked to the strategic direction that appears from the strategic process. A strategy that is congruent, accommodating, and cohesive of innovation is just as important as dynamic capabilities (Gupta & Gupta, 2019; Rajapathirana & Hui, 2018). Therefore, dynamic capabilities need to be developed and implemented mutually while capabilities and strategy can be analytically separated.

Here, it is worth mentioning that this domain of knowledge has received considerable attention of both practitioners and academics in the last 20 years. However, much of the work has been conducted in the context of adaptability, alignment, resilience, and agility. Although the recent evidence has highlighted the need of dynamic capability variables in different contexts, the role of these resources has received scant attention and has not been examined for organizational agility (Dubey et al., 2021). Further, a broad review of the existing literature will substantiate an apparent lack of understanding and evidence regarding the concept and dimensions of dynamic capability (Feizabadi et al., 2019; Tan et al., 2015). Thereby, researchers need to, comprehensively, investigate theories regarding any causal associations anticipated between practices, performance, and capabilities by examining the indirect effect of other contextual factors. In addition, there is a significant gap in the portrayal of the particular procedures integrated by IT managers for learning internal business requirements and codifications (Kar et al., 2021). Further, the topic of IT capabilities and their impact on the performance of the IT sector in developed countries is extensively debated (Brusset & Teller, 2017; Yu et al., 2018). However, limited studies have focused on the role and prominence of dynamic capabilities with reference to organizational agility in settings and circumstances that represent emerging economies like the Middle East (Yu et al., 2018; Zhan et al., 2018).

It seems rational to state that the association between various aspects of dynamic capabilities (sensing, seizing, and transforming capabilities) and organizational agility follows concomitant paths and reflects implicit mechanisms. Therefore, we evoke IT capability to serve as a mediator between dynamic capabilities and organizational agility. In this essence, the integration of technical resources inside and outside an organization is primarily influenced by the IT infrastructure (Adikari et al., 2021). For instance, there is a significant reduction in the operational costs due to rapid growth of cloud computing, which is beneficial for IT firms, as they can integrate technical resources using cloud-based shared resources (Bruque-Cámara et al., 2016; Taghavifard & Majidian, 2022). A firm with adequate IT infrastructure will be more capable to strengthen the association between supply chain capabilities and organizational agility. Thereby, IT firms become more responsive and adaptive as their infrastructures develop foundation of information in the supply chain to fulfill the operational needs (Mikalef & Pateli, 2017; Shukla, Sushil and Sharma, 2019). Further, the operational performance and supply chain visibility are expected to be enhanced based on inter-organizational enterprise information systems (Liu et al., 2013). Along similar lines, the agile performance of IT firms depends on the reinforcement of Internet-enabled technologies (Yusuf et al., 2004). Advanced sets of IT capabilities are promoted based on the IT infrastructure. Previous studies have also treated IT infrastructure as the foundation that enables coordination of operations across the supply chain via the integration and synchronization of information and improvement in responsiveness toward customers (Adikari et al., 2021; Kushwaha & Kar, 2020a, 2020b; Kushwaha et al., 2020; Lee & Whang, 2004; Lu & Ramamurthy, 2011). That is, alignment holds substantial importance in improving firm performance (Shukla et al., 2019). Therefore, it has become yet another front to be achieved in the IT sector (Cheng et al., 2018). Taking the above together, it seems theoretically rational to propose a mediating role of IT capabilities on the dynamic–agility association.

Therefore, this study aims to fill the gaps found in previous studies and add to the current state of knowledge on this domain of organizational agility by investigating the impact of dynamic capabilities on organizational agility, along with examining the mediation influence of IT capabilities. The study also outlines different environmental settings facilitating worthiness of IT-enabled dynamic capabilities. The majority of the previous research attempts have concentrated on examining the attributes of agility (Appelbaum et al., 2017a, 2017b; Gao et al., 2020). Flexibility, culture of change, speed, integration and low complexity, mobilization of core competencies, responsiveness, high-quality and customized products have been considered as attributes or characteristics of organizational agility in previous studies (Sherehiy et al., 2007). This study examines whether these dynamic capabilities are substantial in the strategic management and business management areas, and whether new sustainability narratives are effective from the perspective of dynamic capabilities to strengthen IT infrastructure at the cluster level. In particular, there is no collective accord regarding the organizational agility features. The fundamental constructs have not been appropriately operationalized as the emphasis of dynamic capabilities has mainly been on the consideration of theoretical underpinnings.

More specifically, the contribution of this study is quadruplicate. Firstly, the study has developed a theoretical framework for studying the association between organizational agility and dynamic capability. This association has not been evaluated in the literary articles with reference to their effects in the IT sector based on dynamic capabilities. Secondly, this study expressively seeks enriching the information technology literature by offering guidelines on the roles of organizational agility in improving firm’s social, economic, and environmental performance. Thirdly, this study assists IT managers to understand the role of different aspects of dynamic agile practices within their organizations from the practitioners’ perspectives. This study assists IT-enabled organizations in inventing new methods and procedures to develop improved organization capabilities according to their rivals. Finally, the knowledge domain of IT capability and its impact on the performance of the IT sector in developed countries is extensively debated and examined (Brusset & Teller, 2017; Yu et al., 2018). However, limited studies have focused on the role and prominence of dynamic capabilities conducive to organizational agility in the context of emerging economies (Yu et al., 2018; Zhan et al., 2018). Thus, establishing a research line and discourse that cover this domain of knowledge in the Middle Eastern professional context seems beneficial.

Based on the above, this study intends to answer the following research questions:

1. Is there any direct relationship between resource-based dynamic capability and IT capabilities?
2. Is there any direct relationship between resource-based dynamic capability and organizational agility?
3. Does resource-based dynamic capability influence organizational agility more through an indirect association (via the mediation of IT capability) than an indirect one?

## Theoretical Background and Hypotheses Development

The advancement of technologies like virtual reality (VR), artificial intelligence (AI), Internet of things (IoT), machine learning (ML), cloud technologies, and data analytics has been rapidly evolving (Taghavifard & Majidian, 2022) and, hence, affecting the labor market. As new technologies boom, the presence of such rare talents and competencies becomes a huge confrontation for organizations to handle in the current orientation toward digitalization. This pushes the companies toward devising ambidextrous strategies through constant internal development of talents to respond to the imminent necessities, while utilizing talents for the ongoing projects. The success of a company’s strategy in enhancing human capital and skills relies on the dexterous behaviors generated by the workers who display high levels of passion toward learning and talent acquisition (Kar, et al., 2021).

Accordingly, IT firms are currently involved in major supply chains that use IT as a cost-effective tool bridging the gap between the components of supply chain (Fainshmidt et al., 2016; Shukla et al., 2019). Capitalizing on resources and best practices of partners might help firms in improving their performance (Liu et al., 2013). The concept of dynamic capability of a firm in resource-based view (RBV) has drawn the attention of many scholars who consider it as a major driving force behind the exceptional performance of a firm (Mikalef & Pateli, 2017). Organizational abilities can be explained as the competence of a firm to manage complex human resources essential for accomplishing a desired corporate performance (Harsch & Festing, 2020). Dynamic capabilities assist IT firms to align and harmonize with the external environment. The main challenge faced by these firms lies in the alignment of operational and dynamic capabilities (Helfat & Winter, 2011). The core objective of the RBV is that the competition of companies should be based on the resources and capabilities (Barney, 2001; Rumelt & Lamb, 1984; Wernerfelt, 1984). Many strategic issues have been investigated influentially and purposefully through this inward-looking approach with respect to the situations that demand diversification (Wright et al., 2001).

The distinctiveness of the current study lies in providing explanation on dynamic capabilities from a hierarchical viewpoint. Superior performance cannot be delivered alone by the IT infrastructure; it is, rather, delivered by the assimilation of supportive functions that include supply chains, warehousing, delivery, and logistics (Yu et al., 2018). The expansion of operational capabilities such as organizational agility and sourcing flexibility is supported by inter-organizational information management capabilities (Bondzi–Simpson and Agomor, 2021; Gao et al., 2020).

### Resource-based Dynamic Capabilities, IT Capabilities, and Organizational Agility

Agility is an important construct thought to enhance IT sector’s performance; it is achieved via the firm’s capability to promote elasticity, vigilance, and full integration of operational and managerial processes and mechanisms (Alzoubi & Gill, 2022; Gao et al., 2020; Harsch & Festing, 2020; Vaishnavi & Suresh, 2020). That is, the firm’s capability to be visible is observed in the firm’s consciousness of the surrounding stimuli, its ability to detect threatening modifications, and favorable opportunities. Accordingly, this is expected to help in fostering supply chain agility regularly that is thought to facilitate exchanging essential information between the firm’s customers and their relative suppliers (Kushwaha & Kar, 2020a). Along similar lines, the virtual integration of information technology seems vital for magnifying the amount of information shared between various stakeholders of supply chain (Shukla et al., 2019). This, in turn, will enable detecting intricacy and reaching the maximum degree of agility despite the firm’s capability in enhancing and maximizing its investments in information technology (Kushwaha & Kar, 2020a; Marin-Garcia et al., 2018).

Furthermore, organizational agility can be attained via integrating and modifying the firm’s operations according to external criteria and gauges (Feizabadi et al., 2019). That is, demand management is playing an important role in enhancing supply chain capabilities (Pérez-Pérez et al., 2019; Tuan, 2016). Here, the firm’s orientation to apply a differentiation strategy on its products and/or services can be effectively and efficiently attained by emphasizing on enhancing its capabilities in demand management (Mokhtar et al., 2019).

Performance is represented as the most frequent explored outcome of supply chain alignment in terms of both adaptability and agility (Dubey et al., 2021; Pérez-Pérez et al., 2019). Several studies have employed an average organizational performance based on operational, social, and cultural aspects (Attia, 2015). For example, it has been reported that sales growth, profit, and ROI measure the effects of supply chain association on firm performance (Attia, 2015; Shukla et al., 2019). Efficiency and visibility are increased by lead times, time-to-market, and reductions in costs through integration of supply chain alignment (Ashrafi et al., 2019). Several studies have differentiated between performance measures as well as investigated the effects of association between operational performance and supply chains (Feizabadi et al., 2019).

In particular, the empirical effects are inconsistent regarding supply chain alignment on performance (Marin-Garcia et al., 2018). The association between supply chain integration and performance has been established through general support (Mokhtar et al., 2019). On the contrary, the association is confirmed merely for innovation and delivery, but not identified through flexibility, cost, and quality in the context of operational perspective (Gunasekaran et al., 2017).

Studies have emphasized on additional particular alignment consequences that include quality, process enhancement, innovation, and sustainability (Attia, 2016). For instance, logistics innovation is related to external quality, whereas introduction of new product to market is associated with supplier integration (Mokhtar et al., 2019). It has been noted that supplier integration is connected with innovation, including of social media platforms, and enhancement in order to associate customer integration (Adikari et al., 2021; Kushwaha & Kar, 2020b; Kushwaha et al., 2020; Singh, 2013). In addition, it has been revealed that collaboration is associated with environmental competitiveness, supply chain sustainability assessment, and confirmation integration (Tuan, 2016).

Innovation is another important outcome of adaptable supply chain, particularly in terms of product life cycles, which have been increased in different industries (Luu, 2019). It has been purported that the ability of an organization for innovation is improved through supply chain adaptability (Marin-Garcia et al., 2018). For instance, it has been argued that there is an association between supplier innovation and adaptive capability of manufacturers (Ashrafi et al., 2019; Gupta & Gupta, 2019). In this regard, organizations are allowed to improve customer value by means of ambidextrous operational abilities through supply chain adaptability (Alfalla-Luque et al., 2018). When capitalizing merely on exploitative activities as well as tendency to lose efficiency, supply chains are subjected to sub-optimality when they are not competent enough to benefit new notions (Nguyen, 2017). Through this ambidextrous strategy, superior market performance is driven by managing this trade-off.

Capabilities are competencies required for developing resources that contribute to achieving preferred objectives through organizational procedures; commodities controlled or owned by the firms are referred to as resources (Peteraf, 1993). These capabilities and resources are tangible or intangible information-based procedures that are developed over time and are specific to a firm. Dominant and distinctive capabilities and resources might become the foundation of competitive edge (Chikhale & Mansouri, 2015; Peteraf, 1993). These resources of a firm need to be valuable, immobile, rare, imperfectly imitable, and heterogeneous (Barney, 2001).

The essence of dynamic capabilities lies in those practices that facilitate firms to respond swiftly in order to modify environments while developing, identifying, reconfiguring, and integrating capabilities and resources (Teece, 2018). The above-mentioned acts might entail product development, strategic planning routines, knowledge creation, etc., in the presence of entrepreneurial innovation and evolutionary modifications (Adikari et al., 2021). In this regard, dynamic capabilities facilitate firms to shape their organizational environment and to adapt to the changing environment. Sensing and shaping have different objectives and might have different micro-foundations that perceive sensing as a means to identify, shape, and create market disequilibrium (Gupta & Gupta, 2019; Kirzner, 2015).

In dynamic capabilities, it is essential to engage both supplies and customers in order to build a comprehensive understanding of their needs, hence following a successful and effective process of decision-making (Mathu & Phetla, 2018). Furthermore, this helps in generating creative ideas and divergent thoughts of customers on the firm’s launch of new products (Kushwaha & Kar, 2020a). Moreover, engaging and involving suppliers and customers require wise and smart usage of dynamic capabilities, which is vital for guaranteeing a rapid response to the unprecedented changes in the surrounding environment (Miraz et al., 2018).

Therefore, it might be advantageous to, independently, consider reshaping innovative or absorptive capacities of an organization (Cohen & Levinthal, 1990; Gupta & Gupta, 2019; McGrath, 2001). They are purely related to internal and external organizational learning, respectively. They need competence for creating new chances internally within an organization. Therefore, dynamic capabilities, which are based on IT resources, are conceptualized as the ability of an organization to effectively alter its course of development in accordance with a business process and in comparison with its rivals in terms of cost reduction (Pérez-Pérez et al., 2019), maximum business learning (Kar et al., 2021; Kushwaha et al., 2021), intelligence, and integration of activities (Adikari et al., 2021; Schwarz et al., 2019). Accordingly, the following hypotheses are presented:

H_1_: Resource-based dynamic capabilities (sensing capability, seizing capability, and transforming capability) are positively associated with IT capabilities.

H_2_: Resource-based dynamic capabilities (sensing capability, seizing capability, and transforming capability) are positively associated with organizational agility.

### The Mediation Role of IT Capabilities

The integration of technical resources inside and outside an organization is significantly affected by the IT infrastructure (Adikari et al., 2021). For example, there is a significant reduction in the operational costs due to rapid growth of cloud computing, which is beneficial for IT firms, as they can integrate technical resources using cloud-based shared resources (Bruque-Cámara et al., 2016; Taghavifard & Majidian, 2022). Digital technologies and industrial Internet of things (IIoT) have been developing rapidly and creating defiance and commotion in guaranteeing the presence of certain key competencies in job profiles. On the one hand, it has become essential to, continuously, develop more contemporary job profiles over the relying on the current ones, which are expected to become outdated and irrelevant due to the dramatic changing demands of IT skills. Professional individuals are faced with the challenges of undesirability and compulsion for constant upskilling, requalifying, and training for future. It is obvious, then, that technological interventions of Internet of things (e.g., robotics, artificial intelligence, augmented reality, and big data analytics) are continuously upgrading; hence, they spark a thorough analysis of professionals’ skills acquisition behaviors (Kar et al., 2021).

A firm with an adequate IT infrastructure is more likely expected to strengthen the association between supply chain capabilities and organizational agility. Thereby, the IT firm becomes more responsive and adaptive, as its infrastructure develops a foundation of information in the supply chain that fulfills the operational needs (Mikalef & Pateli, 2017).

Integration of technical resources in the organizational management research community has gained a significant attention (Adikari et al., 2021). The operational performance and supply chain visibility is enhanced based on inter-organizational enterprise information systems (Liu et al., 2013). This shows the need of investigating the association between IT capabilities and organizational agility to expand our vision beyond the internal resources. For instance, Gao et al. (2020) conducted a study examining how the interaction with two axial features of IT capabilities (spanning and flexibility) can affect organizational agility. More specifically, the study found that the association between IT business spanning capability and IT flexibility was mutually positive. However, along opposite direction, the association among the features of IT integration and spanning was found mutually negative.

The agile performance of IT firms depends on the reinforcement of Internet-enabled technologies (Yusuf et al., 2004). Advanced sets of IT capabilities are promoted based on the IT infrastructure. A study conducted by Tiwari et al. (2015) states that a flexible IT infrastructure plays an important role in managing operations under environmental turbulence and dynamism. Previous studies have also shown IT infrastructure as the foundation that enables coordination of operations across the supply chain through the integration and synchronization of information and improvement in responsiveness toward the customers (Adikari et al., 2021; Kushwaha & Kar, 2020a, 2020b; Kushwaha et al., 2020; Lee & Whang, 2004; Lu & Ramamurthy, 2011). Alignment holds substantial importance in enhancing firm performance. Therefore, it has become yet another front to be achieved in the IT sector (Cheng et al., 2018).

The applicability of the concept of alignment is predestined in a wide range of fields, as it is the basis for organization–strategic alignment (Dubey et al., 2021). The alignment of IT infrastructure approaches with the organization’s strategic goals has been fruitless and ineffective due to the organization’s declining performance (Feizabadi et al., 2019). According to Marin-Garcia et al. (2018), the organization’s intra-internal alignment extremely differs from its inter-external alignment. The core focus of alignment is based on its external features due to its representations between different IT actors (Wang & Dass, 2017). The IT members must rely on adaptability to operate more efficiently in today’s dynamic environment. The challenges of constant evolution in demands occur due to the disruptive events that are met through a flexible supply network (Pérez-Pérez et al., 2019; Wang & Dass, 2017). The performance of the relief activities is usually considered as an important platform for success (Barney, 2001).

In the field of information systems, there is a significant impact of IT investments on sustainability of an IT firm (Kushwaha et al., 2020; Wade & Hulland, 2004). The IT firm relates to the strategic movements, IT capabilities, sustainable competitiveness, and long-term impact of IT investments (Wheeler, 2002). Increased attention is being paid on the dynamic business environment, in comparison with the organizational IT resources. In a similar context, organizational agility is one of the IT-enabled intermediate outcomes that has recently drawn the attention of researchers and entrepreneurs. The IT firms are capable enough to detect changes, alter their market strategies, and react accordingly via the digitized platforms that include enterprise resource planning, Internet computing, advanced systems, efficient management of their supply chain, and interaction with customers. Such firms are also able to form healthy collaborations with their partners essential for dealing with the emerging markets. Thus, it can be stated that organizational agility helps to streamline work processes and builds inter-organizational relationships (Agarwal & Sambamurthy, 2002).

The significance of information technology lies in its capability to enhance the firm’s performance via establishing a database and a podium facilitating interaction, exchange of information, and partnership between various involved entities (Aslam et al., 2018; Kushwaha & Kar, 2020b; Kushwaha et al., 2020). Thus, such a domain is thought to facilitate the isolation of the individual from that affected domain; hence, it accordingly minimizes the effect of a disturbance on the internal cohort (Bidhandi & Valmohammadi, 2017). Use of information technology cannot be limited to a specific kind of task and action; instead, it includes the operational and performance procedures, activities, systems, personnel, and the surrounding climate (Cheng et al., 2018). Moreover, information system enables an organization to integrate strategies for rapid decision-making. This aspect additionally enhances the organizational performance in terms of its elasticity (Dubey et al., 2021; Kushwaha et al., 2020). Thus, based on the above discussion, the following hypothesis is proposed.

H_3_: IT capabilities (IT infrastructure and IT investments) mediate the association between resource-based dynamic capability and organizational agility.

## Methodology

### Sample and Procedure

Guided by the key objective of this study, it was essential to select the sample from a sector with great emphasis on innovations. Thus, there is a collective agreement among scholars and professionals in that tech-intelligence-based industries face a hyperkinetic competition that forces them to respond to their rivals in very rapid and flexible manners (Bondzi–Simpson and Agomor, 2021; Pérez-Pérez et al., 2019). Toward that end, the ministry of industry and information technology of Jordan was contacted seeking access to the database of firms using high–medium technology. An inviting email has been circulated to participants, facilitating their participation via following a link that leads to the online survey.

More specifically, a cover letter was attached to the self-administered questionnaire to introduce the research objective and explain the significance of participant’s cooperation. The questionnaire was not translated into Arabic (the local language used in Jordan) as the participants in the targeted population (Jordan) were experts working in information technology unit and/or supply chain department and English was their official language.

Nevertheless, language and industry experts thoroughly crosschecked the wording of questions throughout the pilot testing process. A 5-point Likert scale was used to measure the responses ranging from 1 (strongly disagree) to 5 (strongly agree). A pretest was conducted on the survey questionnaire that involved ten information management experts of IT firms. Their feedback assisted in expurgating the measures and omitting the inappropriate items. Afterward, necessary changes were made in the format and wording of the questionnaire to enhance the content validity of the used measures (Flynn et al., 2010; Huo et al., 2014).

Previous studies often used only one participant per organization. However, dynamic capability and organizational agility is a complicated phenomenon that affects IT company. Therefore, comprehensive answers to all questions cannot be obtained from only one individual. In this regard, different insights were extracted throughout the hierarchy of the firm regarding its consequences, practices, and competence by involving two participants of each company as recommended by Ekrot et al. (2016). Total 600 questionnaires were sent to the sample companies from January to June 2021 and 290 responses were received. The responses included 270 (representing a response rate of 45%) usable responses and 20 invalid responses. The demographic profile of the participating companies is illustrated in Table 1.

**Table 1:** Demographic profile of the participating firms

Item	Measure	*N* (%)
Firm size	< 200 employees	37 (27.1)
	200–500 employees	52 (38.5)
	> 500 employees	46 (34.4)
Firm age	< 2 years	33 (24.8)
	2–5 years	57 (42.2)
	> 5 years	45 (33.0)

### Measurement Instruments

The questionnaire was divided into the following constructs:

Resource-based dynamic capability: The items on resource-based dynamic capability were adopted from Lee and Rha (2016). These items were based on sensing capability, seizing capability, and transforming capability.

IT infrastructure: IT infrastructure was assessed by adopting several items from the well-established scale of Liu et al. (2013), Rai et al. (2006), and Saraf et al. (2007). These items allowed the researcher to ask the participants questions regarding the compatibility of owned IT resources and those deployed for streamlining the operations of their organizations.

IT investments: The items on IT investments allowed the researcher to ask participants questions about the extent of IT investments of their organizations and its use in operational activities of the organizations.

Organizational agility: Organizational agility was evaluated in terms of a firm’s timely response to the internal and external environmental changes. These items were adopted from Mikalef and Pateli (2017) and Rai and Tang (2010).

Control variables: The variables of firm’s operations, firm’s size, and firm’s age were controlled, as the proposed associations might differ to these demographic characteristics of firms. A dummy variable was created for industry operations that had two categories: representing manufacturing operations and indicating service operations. The firm’s age was assessed in years, which was calculated by considering the time of its establishment, while the firm’s size was assessed based on the number of employees.

### Analytical Tools

**Fig. 1:**
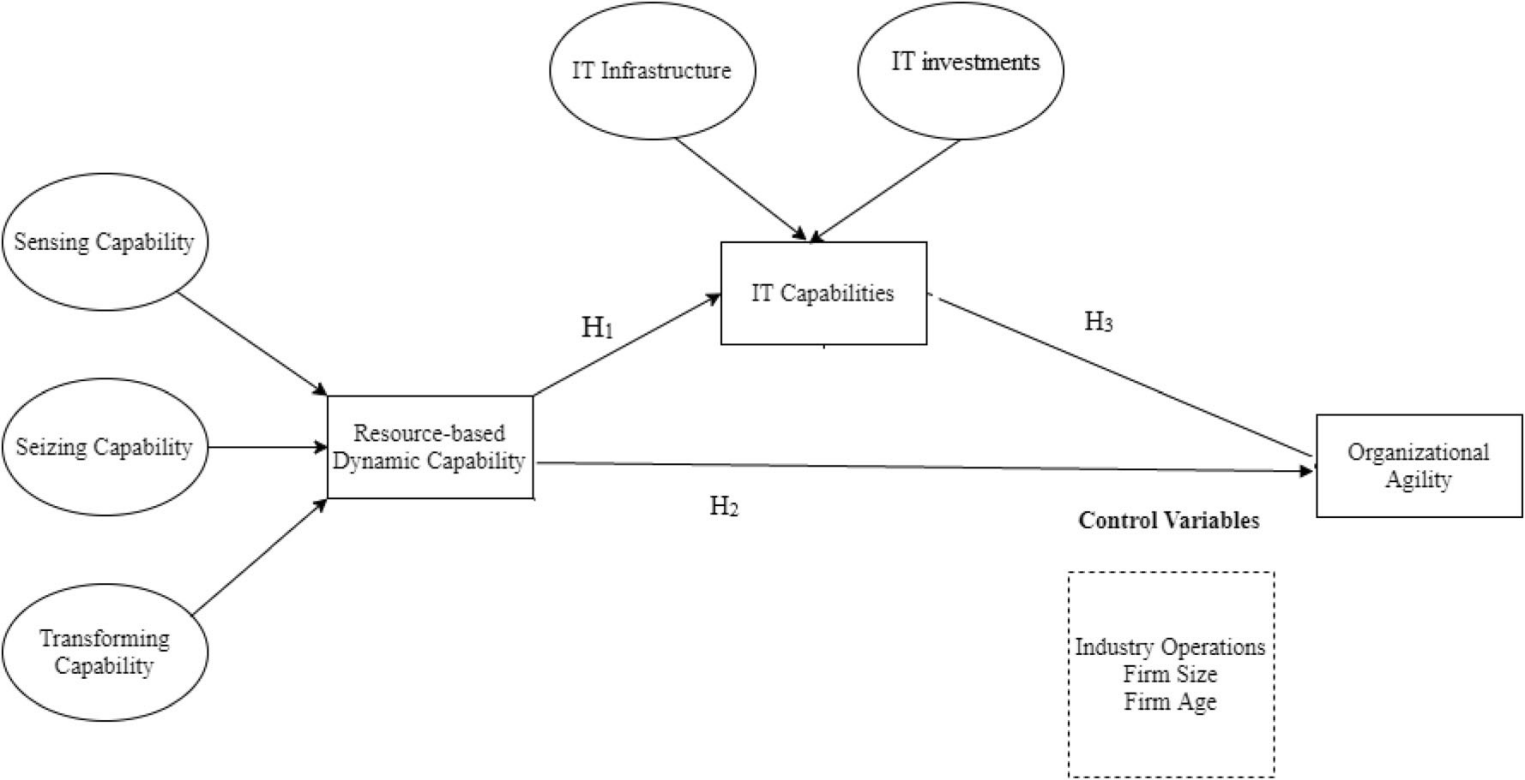
Theoretical framework

PLS-SEM was employed to analyze and interpret the proposed associations among the study variables (dynamic capabilities, IT capabilities, and organizational agility). This choice is due to PLS-SEM functioning ability to specify, estimate, assist, and validate the study model (see Fig. 1). Initially, model fit was tested via the development and validation of the associations among the observable variables and their measurement determining factors and indicators. Consequently, the data were loaded into the structural equation model to examine the association among the endogenous variables.

## Results

### Demographics

The final number of participants was 270 employees representing 135 SMEs (see Table 1). Among these 270 employees, the majority of the employees worked for firms of 200–500 employee size (38.5%) of 2–5 years of organizational tenure (42.2%).

### Validation of Measurements

A two-step approach was followed to examine inner–outer measurement and structural model. Initially, purifying the measurement items that might contaminate the factorial structure of the used constructs due to their low factor/item loadings was achieved using exploratory factor analysis. Accordingly, the factor–item loadings ranged from 0.56 to 0.87, accompanied by t values spanning from 7.99 to 26.21.

The variance inflation factor (VIF) was estimated for examining the potential effect of multicollinearity on the current data set. The test revealed that the highest value recorded was 2.53, which is obviously smaller than 3.3 break off. Hence, it can be declared that multicollinearity was not a concern for the current data set. Similarly, the internal consistency, composite reliability, and average variance extracted (AVE) of the measurement variables were satisfactory reporting values spanning from 0.70 for Cronbach’s alpha, 0.72 to 0.93 for composite reliability, and above the 0.5 break off for AVE.

The association between shared variance among the average variance extracted (AVE) and other constructs was analyzed for evaluating the discriminant validity, following Fornell and Larcker’s (1981) criterion. Table 2 illustrates the AVEs’ square roots for all the variables. The results suggest no concern of discriminant validity in the research framework, as the correlation values of all the variables were higher than the 0.5 threshold (Fornell & Larcker, 1981).

**Table 2:** Measurement model with values of discriminant validity

Variable	CITC	Alpha	1	2	3	4
IT infrastructure	0.57	0.82				
IT investments	0.66	0.83	0.52**			
Resource-based dynamic capability	0.59	0.82	0.46**	0.37**		
Organizational agility	0.61	0.86	0.41**	0.51**	0.39**	
Mean			4.27	4.19	4.33	4.31
SD			0.34	0.55	0.39	0.51

*p < 0.05, **p < 0.01, ***p < 0.001

The results showed that the variance on the dependent variable (organizational agility) is significantly (*p* < 0.001) predictable by the independent variable (resource-based dynamic capability), obtaining an explanatory power (*R*^2^) value = 0.46. Additionally, no significant variation was detected for explanatory power of the control variables (firm age, operations, and size) expected to have on the model paths. The results reflecting the direct associations of the theoretical framework are illustrated in Table 3.

**Table 3:** Testing the direct paths via linear regression analysis

Path	Structural paths	Coefficient	*p* value	Status
Path 1	Resource-based dynamic capabilities IT capabilities	0.13	0.07	Not supported
Path 2	Resource-based dynamic capabilities organizational agility	0.15*	0.003	Supported
Path 3	IT capabilities organizational agility	0.48***	0.000	Supported

*p < 0.05; ***p < 0.001

### Mediation Analysis

An orienteering procedure was used to test the mediating effect in the structural path model. Initially, the direct path that links dynamic capabilities to organizational agility was assessed. Accordingly, it was found that the association between dynamic capabilities and organizational agility has been minimized due to the effect of other posterior routs such as IT capabilities. In terms of evaluating the indirect influence between the study variables, we draw on Hayes and Scharkow’s (2013) approach, setting the confidence interval of 95% along with 2000-times bootstrapping. Table 4 illustrates the indirect path coefficients and significance levels for all paths included in the theoretical framework. More specifically, the analysis, using PLS-SEM, revealed that a significant mediating influence of IT capabilities on the dynamic capabilities–organizational agility relationship.

**Table 4:** Estimation of total effects via path analysis (multiple regression)

Indirect path	Lower bound	Upper bound	*p* value
Resource-based dynamic capabilities IT capabilities organizational agility	0.13	0.37	0.000***
IT capabilities organizational agility	0.52	0.73	0.000***
Industry operations resource-based dynamic capabilities	0.24	0.36	0.000***
Industry operations IT capabilities	0.15	0.41	0.000***
Firm size resource-based dynamic capabilities	0.24	0.54	0.372
Firm size IT capabilities	0.19	0.61	0.043*
Firm age resource-based dynamic capabilities	0.38	0.67	0.000***
Firm age IT capabilities	0.87	0.62	0.000***

*p < 0.05, **p < 0.01, ***p < 0.001

The key objective of the current study revolves around explicating the nature and magnitude of the associations between dynamic capabilities and organizational agility. The current findings corroborate what has been found in the agility literature (e.g., Huo, 2012; Radhakrishnan et al., 2018; Yu et al., 2018) in that dynamic capabilities and organizational agility are interrelated. However, the first hypothesis (H1) proposing a positive association between resource-based dynamic capabilities and IT capabilities was not supported in the current data set. This aligns with Liu et al.’s (2013) findings, where the dynamic capability–organizational agility interrelation was affected by a full mediation. Thus, the current findings lend additional support to the notion stating that firms need to intensively adopt IT in its operational, quality assurance, and managerial systems, especially for companies operating in developing countries and suffering from a lack of technological integration.

## Discussion

### Summary of Findings

Based on the first research question, this study found that dynamic capability is a catalyst in enhancing the firm’s activities and awareness of the changes in the strategic and business management. The findings indicated that the actual performance of organizational agility was less than dynamic capabilities but greater than knowledge management and innovation. Dynamic capabilities were positively associated with organizational agility. In context with the second research question of the study, the findings emphasized that dynamic capability was a fundamental factor in recreating better services and sustaining a constantly thriving business environment. Finally, in light with the third question pertaining the imperative effect of IT capabilities on organizational agility, the findings indicated a mediating role IT capabilities can play between dynamic capabilities and organizational agility. Therefore, firms should prefer market-associated changes, rapidly balance changes in demand from the market, and deliver the request of customers. The practical implications and managerial interventions that can be stemmed from these findings are discussed in the “managerial implications” section.

In an attempt to answer the developed research questions regarding the direct associations between dynamic capabilities, IT capabilities, and organizational agility, this study has presented both theoretical and practical implications. Initially, it showed viewpoints on how dynamic capability, along with organizational capabilities and resources, influences and improves organizational agility. Dynamic capabilities can develop values under appropriate conditions. The values of dynamic capabilities can be influenced by IT capabilities. Thus, IT-enabled dynamic capabilities should be explored as a segment of a big picture in order to completely realize the advantages of IT resources.

### Theoretical Implications

As mentioned earlier, the resource-based view (RBV) has evoked the firm’s dynamic capabilities as a key driving force responsible for generating exceptional performance of a firm (Mikalef & Pateli, 2017). In alignment to that theoretical perspective, the findings of this study emphasize that organizational abilities can be explained as the competence of a firm to manage complex human resources in order to accomplish the desired corporate outcomes (Harsch & Festing, 2020). Further, this study expands the scope of RBV by including other underlying and implicit forces (the mediation of IT capability) to the dynamic–agility path. This amalgamation of dynamic capabilities with IT capabilities will assist IT firms to align and harmonize with the external environment. The main challenge faced by these firms lies in the alignment of operational and dynamic capabilities (Helfat & Winter, 2011). The core role of the RBV-related concepts, then, is to provide an influential and purposeful inward-looking approach that considers the role of the firm’s IT capabilities in enhancing its use and management of resource-based dynamic capabilities (Wright et al., 2001). Therefore, this study is one of the few empirical studies that devote attention toward the significance of IT-enabled dynamic capabilities to argue that IT can assist a firm in increasing its strategic values by improving organizational agility. This study provides opportunities to conduct further empirical research on IT-enabled dynamic capabilities and organizational agility; hence, it stands as a pioneering research that investigates the impact of dynamic capabilities from the perspective of organizational agility. For instance, it points out on how further research is urged to investigate approaches on applying, utilizing, and handling IT-enabled dynamic capabilities in reference to improving organizational agility.

Further, this study also recommends that IT infrastructure should be viewed as a strategic element for organizations, theorizing that the responding dimension of organizational agility can be facilitated by IT capabilities via IT infrastructure. It is claimed that a flexible IT infrastructure is an important aspect to respond to the organizational capability, which is based on the framework of dynamic capability. It is obvious from the findings that IT capabilities hold the status of a major factor developing organizational agility and consequently have a direct effect on organizational performance (Lee et al., 2008). Thereby, IT capabilities and IT infrastructure have strategic values. Therefore, it is recommended that future studies should examine and evaluate new ways and approaches for building a flexible organizational agility.

Furthermore, several concepts have been clarified by this study, which were not well articulated in the previous studies conducted in the domain of information system. Finally, this study has extended the current work on IT-enabled dynamic capabilities by offering insights on how IT capabilities can be incorporated and applied to enhance the capability of an organization to be more agile (Chikhale & Mansouri, 2015). This study calls for carrying out further studies devoting extra emphasis on information systems and their characteristic effects. It further calls for unlocking the hidden aspects of an increasingly essential but complicated set of relationships between IT resources and organizational agility.

### Practical/Managerial Implications

The current study offers practical suggestions expected to assist firms’ ability in countering market changes inimitably. In a very practical tone, this study suggests that organizations in general and IT firms in particular need to align their technical resources with their strategic business units (Gao et al., 2020). More specifically, the study findings have reflected a positive influence of infrastructure and digital investments on dynamic capabilities that affect organizational agility. IT capabilities significantly and positively mediated the relationship between resource-based dynamic capability and organizational agility. This implies the need for managers to comprehend the concept of dynamic capabilities in order to design plans that are specifically formulated for boosting competitive performance (Curtin et al., 2007; Gao et al., 2020). This involves capturing the right opportunity, identifying the key factors responsible for enhancing growth, and sensing incoming openings (Harsch & Festing, 2020). With dynamic capabilities, firms will be able to satisfy customers’ requirements, effectively interact with their partner firms, and assess internal routines (Adikari et al., 2021; Kushwaha & Kar, 2020a, 2020b).

The findings have also indicated that a stable organizational agility instantly improves services and routines, and this recommends that enhanced services should be given the optimum preference for customer satisfaction (Adikari et al., 2021; Kushwaha & Kar, 2020a, 2020b). In addition, firms should be adaptable enough to easily adjust and respond to unanticipated external modifications. More specifically, knowledge management and industrial intelligence are fine innovations that enable managers to seize meaning from the collected data that can be used create massive data warehouses (Chen et al., 2012). These giant data repositories allow companies to store and detect the significant and relevant changes in customers’ interests, trends, preferences, and purchase behaviors. Thus, the organization’s ability to be agile and responsive to market changes is further enhanced by its IT capabilities (Chikhale & Mansouri, 2015). This, in turn, will help in making accurate decisions that further promote organizational effectiveness and efficiency (Curtin et al., 2007).

### Limitations and Future Research

Several limitations have been identified in this study. Initially, a cross-sectional study design was used in this study, which might offer limitations in terms of the causality associations or time effects between study variables. Further, the data used in this research were gathered throughout a single country (Jordan). Thereby, findings of the study can be subjected to and limited to the specific attributes of the Middle East. The generalizability of the study, therefore, can be improved with utilizing additional data sets obtained from different cultures and regions. The findings revealed by the present study may recommend that an IT-enabled firm can develop different types of IT capabilities correspondingly; hence, they call for additional investigation and an in-depth level of theorizing in this domain of research.

Further, the associations between dynamic capabilities, IT capabilities, and organizational agility are complex and expected to connote nonlinear mechanisms and research veins. That is to say, the current model can be expanded to include some moderating factors (e.g., organizational culture) that can enhance our understanding of the nuanced mechanisms thought to foster organizational agility. For instance, certain organization’ cultural archetypes have been found to facilitate a successful and effective implementation of total quality management interventions (Ababneh, 2020). Along similar theoretical path, organizational culture is, therefore, expected to moderate the associations explored in the current study. Thus, future research is strongly encouraged to draw on Cameron and Quinn’s (1999) model when examining the moderation effect of organizational culture on the theoretical mediation associations between the variables of the current study.

Further, firms can exploit market opportunities to improve swiftness of internal operations with better organizational agility. The findings revealed that dynamic capabilities facilitate organizational agility and competitive performance in a turbulent business environment. This study also offers a parameter for managers to discuss with their relative stakeholders how to manage and use IT capabilities throughout the firm.

## Conclusion

This study suggests a novel progression to the current research endeavor exploring the influence of IT capabilities on organizational agility in the IT sector. More specifically, the current study has used the perspective of dynamic capability to explain the significance of organizational agility in the IT sector. The study suggests that dynamic capabilities of IT firms play an important role in identifying the value of IT resources, which needs to be mobilized across the value chain. In the contemporary business world, the IT sector needs to be equipped with necessary technical resources required to integrate strategic business units with their stakeholders (Gao et al., 2020). Toward that end, professionals were approached from the supply chain management and operational departments of IT firms operating in Jordan. The study findings indicated positive associations between infrastructure, digital investments, dynamic capabilities, and organizational agility. More precisely, IT capabilities significantly and positively mediated the relationship between resource-based dynamic capability and organizational agility. This implies the need for managers to understand the concept of dynamic capabilities in order to design plans that are formulated for boosting competitive performance (Curtin et al., 2007; Gao et al., 2020). This also involves seizing the right opportunity, identifying the major factors for enhancing growth, and sensing incoming openings (Harsch & Festing, 2020). With dynamic capabilities, firms will be able to satisfy customers’ requirements, effectively interact with their partner firms, and assess internal routines (Adikari et al., 2021; Kushwaha & Kar, 2020a, 2020b).

The findings also indicated that a stable organizational agility instantly improves services and routines, which recommends that enhanced services should be given the optimum preference for customer satisfaction (Adikari et al., 2021; Kushwaha & Kar, 2020a, 2020b). In addition, firms should be adaptable enough to easily adjust and respond to unanticipated modifications. More specifically, knowledge management and industrial intelligence are key innovations that enable managers to seize meaning from the collected data that can be used to create massive data warehouses (Chen et al., 2012). Thus, the organization’s ability to be agile and responsive to market changes is further enhanced. This, in turn, will help in making accurate decisions that further promote organizational effectiveness and efficiency (Curtin et al., 2007).

Key Questions Reflecting Applicability in RealLife

1. Is there any direct relationship between resource-based dynamic capability and IT capabilities?
2. Is there any direct relationship between resource-based dynamic capability and organizational agility?
3. Does resource-based dynamic capability influence organizational agility more through an indirect association (via the mediation of IT capability) than an indirect one?
